# Acute Pancreatitis, with Report of a Case Successfully Diagnosticated and Operated

**Published:** 1914-09

**Authors:** Ross G. Loop

**Affiliations:** Elmira, N. Y.; Attending Surgeon, Arnot-Ogden Memorial Hospital. 359 Main St.


### Acute Pancreatitis, with Report of a Case Successfully Diagnosticated and Operated.

By ROSS G. LOOP, M. D., F. A. C. S., Elmira, N. Y.

Attending Surgeon, Arnot-Ogden Memorial Hospital.

From the paucity of the literature one must conclude either that acute pancreatitis is a very rare disease or that we are failing to recognize it. Probably both deductions are in part correct. In the light of the experience which I have had in the case to be reported, looking back after sixteen years of fairly active work in the surgical emergencies of the abdomen, I can recall no case resembling this which was overlooked. I also note that many operators of wide experience report only two to five or six cases. On the other hand, we quite frequently hear of sudden and violent abdominal symptoms, fatal in their results, and I have no doubt that many cases of acute pancreatitis are buried with the epitaph, ''acute indigestion.''

Reasoning by analogy, one might expect acute infection of the pancreas to occur as often and as disastrously as acute infections of other appendages of the intestinal tract. The orifice of the pancreatic duct is as open and unguarded as is the choledochus or the appendix. Why then, do not the flora of the bowel as frequently produce destructive inflammation in the pancreas as in these? The studies of Welch and others indicate that bacteria play but a secondary role in pancreatic disease and that in the acute types, we are dealing with a chemical and not a bacterial process. In fact, bacterial growth in the pancreas is apparently in inverse proportion to the secreting power of the gland.

This raises the question of an inherent protection of the pancreas against bacterial invasion. Are the various enzymes of its secretion bactericidal? Physiologists have demonstrated that of its three ferments, two, amylopsin and steapsin, are secreted in active form, and attack respectively carbohydrates and fats, but that the third, trypsin, the one of all which might attack and destroy bacteria, is secreted as trypsinogen, whose powers are latent until activated by secretin, a substance produced by the action of the acid chyme on prosecretin contained in the duodenal mucous membrane, and conveyed by the blood to the pancreas.

Assuming then that trypsin does digest bacteria and thus afford protection to the pancreas, why can not infection occur between meals when, as we are told, trypsin is in its inactive form of trypsinogen? This raises another question, does excessive bacterial growth in the intestine produce and maintain an acidity of kind and degree sufficient to keep trypsinogen constantly activated and thus always on guard? Answers to these questions might throw light on the apparent immunity of the pancreas to infection and on the etiology of a small percentage of the cases of acute inflammatory processes in it, not explained by the generally accepted calculus theory.

The importance of biliary calculi in the causation of acute pancreatitis was first pointed out, I believe, by Opie, and his observations and experiments, confirmed by many others, are now accepted facts. He has showed how a calculus of the right size and shape, may lodge in the ampula of Vatter in such a way as to divert the flow of bile into the pancreatic duct and by further experiments, he has demonstrated the destructive effects on pancreatic tissue of the bile so diverted. In animals killed at varying times after injections of bile into the pancreas, the conditions found indicate that at first necrosis occurs, involving both parenchyma and stroma followed by hemmorhage from vessels whose walls have been damaged and that bacterial invasion, if it ensues at all, as well as gangrene, are secondary to the changes first mentioned. I do not find many supporters for the theory of primary hemorrhage, a sort of pancreatic apoplexy, although a few do adhere to this idea. The rapid and profoundly destructive effects of bile, ordinarily so innoxious a fluid, on the pancreas, suggests that it may have an activating effect on trypsinogen, which as trypsin, may be in whole or in part, the destructive agent. So far as I can find, no experiments have been made showing the state of this ferment during the course of acute pancreatitis.

So far then as our present knowledge goes, acute pancreatitis is a chemical destruction of the gland with secondary hemorrhages more or less massive, even to the degree of rupture of the gland, infection and gangrene following in some cases. It is caused by any agent which activates trypsinogen while yet within the confines of the gland and while this may be brought about by more than one means, the only demonstrated way is by the diversion of bile into the pancreatic duct, usually by a biliary calculus.

The symptoms and diagnosis have not been altered or enlarged to any noteworthy degree since the lecture of Fitz in 1889, which Osler calls "a crystalization of our knowledge of the subject." So typical were these in the case to be reported that I shall dwell upon them even at the risk of reiteration, for I find no condition of the abdomen with which the average practitioner is less familiar than this. He seems to have a confused notion of glycosuria and fat in the stools; that the disease doubtless does occur but that he has never encountered it. Given a soft, glandular organ, with various avenues for infection, producing powerful enzymes, themselves capable of great harm, can it be doubted that acute pancreatitis is more prevelant than is commonly supposed?

We have first, as a rule, pain in the abdominal zone between the costal borders and the level of the umbilicus. It is frequently situated chiefly or wholly at the outer border of the left rectus at the level indicated and in its location, character and radiation, may closely simulate left renal colic. All writers emphasize the severity of the pain and based on my case, this is a most important diagnostic point, it being well-nigh uncontrollable and unbearable. It is usually of sudden onset. Pain is followed, rarely preceded, in a few minutes, by vomiting, likewise persistent. The severity of these two symptoms will ordinarily lead to the summoning of a physician who will be able to observe the further development of the case. He will find little or no disturbance of pulse or temperature and as a rule these functions will be but little disturbed during the progress of the disease. Soon, distention of the upper zone of the abdomen will develop and according to many observers, this phenomenon should always excite suspicion of acute pancreatic disease. There is extreme tenderness over this area and I believe it is a rule that while pain may be most marked on the left, tenderness is greatest over the right rectus. Constipation is marked but total obstruction does not obtain, both flatus and feces rewarding efforts to move the bowels, but without the relief of symptoms which would be expected were the case one of acute intestinal obstruction, which acute pancreatitis most closely simulates. The facies is expressive of pain, anxiety and the gravest danger and the skin assumes a palid, dusky hue. No reliance whatever can be placed on glycosuria or lipochezia (fat in stool). Given these symptoms, especially with a history indicative of gall-bladder disease, a diagnosis of acute pancreatitis is at once warranted.

Differentiation must be made from several emergencies of the upper abdomen but if all cases are as typical as the one appended, a thorough appreciation of the few symptoms above enumerated should indicate the diagnosis. Most closely simulating it is acute intestinal obstruction. The distinctive symptom is that while the bowels are obstinately locked, they are not absolutely so and high enemata, eserine, etc., will invariably produce some result, but **without** any relief of the pain or distention. Messenteric thrombosis will be with difficulty differentiated but in this condition, after the first sudden and severe onset there is usually a remission of symptoms for a few hours or possibly a day or two; obstruction of the bowels is complete and dark blood is usually obtained with the fecal matter washed from the lower bowel.

The other emergencies in this region, such as perforation of a gastric or duodenal ulcer, gangrene or rupture of the gallbladder or bile duct, these produce a local peritonitis and it is to this that the symptoms are mainly due. We have here first the history pointing to the usual antecedents of these conditions, the thermic and circulatory disturbances that result from peritonitis, and above all, the pain in the cases I have seen, at least, is not comparable to the pain of acute pancreatitis. I can give no explanation for this over-shadowing symptom except that offered by Schmidt and others, which is after all far from satisfactory for it might be with equal truth applied to all the viscera of the upper abdomen, viz., its intimate association with the nervous system in general and the solar plexus in particular. It is neuralgic in character, is aggravated by the slightest movement or lightest touch, and while usually felt a little to the left, may be felt on the right or even in the hypogastrium.

The prognosis of acute pancreatitis is most grave, the mortality of reported cases prior to the past five years being from 60% to 100%, and the gloomy attitude of the profession was well voiced by the late Maurice H. Richardson in his review of the subject before the Clinical Congress of the Surgeons of North America at its Philadelphia meeting in 1911, when he said:—"The rarity of successful operation upon an accurately diagnosticated acute lesion of the pancreas is so great that, for practical consideration, these acute infections are almost beyond the reach of successful surgery. They are not beyond our reach in theory; in practice they are so frequently beyond our reach that there is little real hope of cure." Similar statements were made by Richardson and others of unquestioned ability at the meeting of the American Surgical Association in 1912, but at this meeting, Hotchkiss reported one successful operation, LeConte, two cases with recovery, Lilienthal, six cases with five recoveries, a mortality of less than 17%, while Korte, in thirty-four operations, had 52% of recoveries. These recent statistics confirm my own convictions that successful surgery can be done in these cases if seen early enough, thus placing the responsibility on the general practitioner, who first sees them.

A rapid operation, invoking all shock-reducing agencies, with thorough drainage, probably best through the anterior abdominal wall, taking particular pains to unite the incision firmly and to protect it from the destructive efforts of contact with the pancreatic secretion, will certainly improve these statistics and I predict that ten years hence, not only will our diagnostic skill show a greater frequency of this disease than is now dreamed of, but a mortality fairly comparable with other abdominal emergencies.

Case report. Seen with Dr. Charles Haase on December 15, 1912. Male, age 46 years. Married. Salesman, traveling.

Habits exemplary. Previous history appears to have no bearing on present condition. There is no evidence of preceding biliary disease except that he has had frequent attacks of "indigestion" with flatulency after eating, but he has always been a hearty and rapid eater.. There is no history of any attacks simulating this one.

At noon, after a heavy, hurried lunch, patient was taken on the street with sudden, severe pain in the epigastrium, followed almost immediately by nausea and vomiting, which, he thought, would relieve the pain. This was not the case, however, and he returned to his home and soon summoned Dr. Haase, who was unable to relieve either, although four or five grains of morphine were given hypodermatically within the next few hours. Cathartics and enemata yielded fairly satisfactory results, both gas and feces being expelled at intervals during the progress of the disease, but without any amelioration of the symptoms.

I saw him about twenty-eight hours after the onset. Pulse and temperature were about normal. The skin was dusky. There was pain at the outer border of the left rectus above the umbilicus, continuous, but with exacerbations, resembling renal colic but not definitely following down the ureter, the most agonizing I have ever witnessed, a cold sweat accompanying the paroxysms. The abdomen was distended and rigid above the umbilicus and tenderness was greatest just to the right of the mediam line.

Our diagnosis lay between acute intestinal obstruction, probably from volvulus, and acute pancreatitis. Against the former was the excruciating pain and the fact that some feces and considerable flatus were passed during the whole course of the trouble, both with and without enemata. We decided to try the effects of eserine and if there was no relief to consider the case as acute pancreatitis and to operate. Two doses were accordingly given at intervals of three hours and there being no improvement, he was hurried to the hospital. Crile's methods of reducing shock were used and an incision made through the left rectus, just outside the median line, the location of the most marked distention. The appearance of numerous areas of fat necrosis both in the omentum and messentery, but not in the fat of the abdominal wall, soon proved our diagnosis. The most accessible route seemed to be below the transverse colon, through the transverse mesocolon, through which could be seen a dark purple color which proved to be dark bloody fluid. The pancreas, about double its normal size, and resembling a bunch of Concord grapes, was incised in several places and both the pancreatic and common ducts palpated for calculi, with negative results. Cigarette drains were introduced into the pancreatic substance and into the omental bursa and the wound closed in the usual way.

We were not annoyed by hemorrhage during our work and the patient was returned to his room in good condition, both pain and nausea being relieved. Convalescence proceeded in a most satisfactory manner for the first week, with good drainage, at which time the skin sutures were removed, the wound being dry and apparently perfectly healed. During the following night, however, it broke entirely apart, owing to the digestive effects of the pancreatic juice, allowing several coils of the intestines to escape into the dressings. This accident necessitated a second administration of ether and closure of the wound, after removal of the digested tissue from the edges and from the omentum and intestines. A broad, flat cigarette was placed on the under side of the wound to guard against a repetition of this mishap.

Convalescence after this was prolonged and marked by great emaciation and digestive disturbances for which the administration of pancreatin did not afford much help. Drainage continued for about three months and many fragments of necrotic tissue, resembling pancreatic structure were exfoliated. This was without doubt the gangrenous stage which Opie and others consider secondary to the hemorrhagic. At no time were glycosuria or fatty stools noted. At the time of writing, about nine months since the operation, recovery seems essentially complete.

In conclusion, sudden, intense pain across the abdomen above the umbilicus, often resembling left renal colic, so agonizing as to be almost uncontrollable and unbearable; vomiting also persistent; distention of this area; dusky skin; pulse and temperature practically normal (is this due to the non-bacterial nature of the attack?); obstipation, but not complete obstruction; this symptom-complex means acute pancreatitis just so surely as right rectus rigidity and tenderness over McBurney's point mean appendicitis and demand an immediate abdominal section and drainage of the pancreas. I believe that this group of symptoms should be memorized by every practitioner of medicine and its significance appreciated and that when this is done, we shall hear less of fatal "acute indigestion" and more of acute pancreatitis, and with far better statistics.

359 Main St.

---

**Faecal Concrement in Appendix Epiploica.** Francis L. A. Graves, Brit. Med. Jour., January 3, 1914, reports a case in a woman aged 68, operated on for gall stones, in which two such concrements, not communicating with the lumen of the pelvic colon, were found.

